# Recovery of Odorants from an Olfactometer Measured by Proton-Transfer-Reaction Mass Spectrometry

**DOI:** 10.3390/s130607860

**Published:** 2013-06-19

**Authors:** Michael Jørgen Hansen, Anders Peter S. Adamsen, Anders Feilberg

**Affiliations:** Department of Engineering, Aarhus University, Blichers Allé 20, Tjele 8830, Denmark; E-Mails: anders.adamsen@agrsci.dk (A.P.S.A.); anders.feilberg@agrsci.dk (A.F.)

**Keywords:** olfactometry, odor nuisance, livestock, EN 13725

## Abstract

The aim of the present study was to examine the recovery of odorants during the dilution in an olfactometer designed according to the European standard for dynamic olfactometry. Nine odorants in the ppm_v_-range were examined including hydrogen sulfide, methanethiol, dimethyl sulfide, acetic acid, propanoic acid, butanoic acid, trimethylamine, 3-methylphenol and *n*-butanol. Each odorant was diluted in six dilution steps in descending order from 4,096 to 128 times dilutions. The final recovery of dimethyl sulfide and *n*-butanol after a 60-second pulse was only slightly affected by the dilution, whereas the recoveries of the other odorants were significantly affected by the dilution. The final recoveries of carboxylic acids, trimethylamine and 3-methylphenol were affected by the pulse duration and the signals did not reach stable levels within the 60-second pulse, while sulfur compounds and *n*-butanol reach a stable signal within a few seconds. In conclusion, the dilution of odorants in an olfactometer has a high impact on the recovery of odorants and when olfactometry is used to estimate the odor concentration, the recoveries have to be taken into consideration for correct measurements.

## 1. Introduction

Odor nuisance from agriculture and industry is often evaluated as the odor threshold according to the European standard for dynamic olfactometry, EN 13725 [1]. This standard defines how odorous samples should be collected in sample bags, transported to the laboratory, and analyzed by trained panelists in an olfactometer within 30 h. When this method is used to characterize an odor source it has to be taken in to consideration that the chemical composition of odor samples could be affected by the sampling method, storage in sample bags and dilution in the olfactometer. The European standard for dynamic olfactometry [1] defines three types of sample bags that can be used, (i) tetrafluoroethylene hexafluoropropylene copolymer (FEP); (ii) polyvinylfluoride (PVF, Tedlar™); and (iii) polyethyleneterephthalate (PET, Nalophan NA™). It has previously been shown that the concentration of several typical odorants from animal houses (reduced sulfur compounds, amines, carboxylic acids, phenols and indoles) decreases during the storage in one or more types of sampling bags, possibly due to sorption to the bag material and diffusion out of the bags [2–6]. For certain compounds, e.g., phenols, the 24 h recovery is <10% [3,4,6].

The European standard for dynamic olfactometry [1] defines that the materials in the olfactometer in contact with the gaseous sample should be polytetrafluoroehylene (PTFE, Teflon), FEP, PET, glass or stainless steel. The effect of the olfactometer on the odorous samples has only been investigated to a small extent. In a study by Hansen *et al.* [7] it was demonstrated that 10–60% of reduced sulfur compounds (hydrogen sulfide, methanethiol and dimethyl sulfide) were not recovered during the dilution in two types of olfactometers constructed by glass and stainless steel/PTFE, respectively. In a study by Beauchamp *et al.* [8] an olfactometer for medical purposes was investigated, and it was shown that the time to reach maximum intensity is dependent on the time the samples are presented to the panelists (pulse duration). According to the European standard the pulse duration can be a maximum of 15 s, but in practice it is often only a few seconds. The typical odor concentrations measured in animal production is often below a few thousand odor units (OU/m^3^) [9–11], and a low recovery of odorants during the dilution in an olfactometer may have a significant influence on the results causing underestimation of the odor concentration. It is therefore of great interest to investigate the recovery of odorants from animal production in an olfactometer designed according to the European standard for olfactometry [1], and how long the pulse duration should be to reach maximum recovery.

The challenge in relation to estimating the recovery of odorants in an olfactometer is the relatively short pulse duration at each dilution step which is often only a few seconds. Proton-Transfer-Reaction Mass Spectrometry (PTR-MS) has previously been applied for measuring volatile organic compounds in different research areas including medical application, food control and environmental research [12], and has also been used to evaluate the fate of odorants in olfactometers [7,8]. The PTR-MS has a high sensitivity and specificity in relation to odorants from animal houses [13–16], and can provide data with a time resolution down to a few hundred milliseconds, which makes it possible to follow the actual concentration of odorants presented to the human nose in the olfactometer. The previous studies regarding recovery and pulse duration [7,8] have only included a limited number of odorants (sulfur compounds, ketones and alcohols), but the air from animal production is much more complex [13,17] and there is a need to investigate the effect on a wider range of odorants.

The aim of the present study was to investigate the effect of various pulse duration on the recovery of selected odorants in an olfactometer fulfilling the European standard for dynamic olfactometry [1]. Typical odorants found in pig production such as sulfur compounds, amines, carboxylic acids and phenols were included, along with *n*-butanol that is used to select panelists for olfactometry.

## 2. Experimental Section

### 2.1. Analytical Instruments

The olfactometer used in the present study was a commercial TO8 instrument manufactured by Odournet GmbH (Kiel, Germany). The olfactometer was based on the yes-no method with four sniffing ports for panelists. The nose cones for the panelist were made of glass and had a conical shape. The dilution system was constructed with gas jet pumps and calibrated orifices for sample dosage. The parts of the olfactometer in contact with the odorous gas mixtures were made of stainless steel (orifices) and PTFE (tubing). Dilution air for the olfactometer was provided by a compressor (Dr. sonic, Fini, Bologna, Italy) and was filtered by a column containing silica gel and charcoal in order to provide dry and odor free air. At each dilution step the diluted sample air was provided to one sniffing port at a time while the others received dilution air. The flow out of the nose cone with diluted sample air was 20 L·min^−1^. The olfactometer was calibrated by Odournet GmbH according to the European standard for dynamic olfactometry [1] prior to the measurements by using propane in nitrogen as a reference gas.

A high sensitivity PTR-MS (Ionicon Analytik, Innsbruck, Austria) was used to measure the concentration of odorants in the gas mixtures prior to dilution and in the olfactometer at each dilution step. The PTR-MS is based on soft ionization with protonated water and subsequent detection in a quadropole mass spectrometer. The principle of the PTR-MS has been described in details in previous studies [12,18]. The PTR-MS was operated under standard drift tube condition with a voltage of 600 V, pressure between 2.1–2.2 mbar and a temperature of 60 °C. The inlet of the PTR-MS consisted of a 1.2 m polyether ether ketone (PEEK) tubing with an outer diameter of 1.6 mm and an inner diameter of 0.064 mm. The inlet temperature of the PTR-MS was 60 °C and the inlet flow was ca. 85 mL·min^−1^. In order to achieve a high resolution in data only the primary ion (protonated water; H_3_O^+^) and one ion for each odorant were measured during the measurements in the nose cone. The determination of concentrations was based on known gas standards or reaction rate constants (trimethylamine and 3-methylphenol). The dwell time was set at 200 ms for the primary ion, 500 ms for the odorants, so one measuring cycle was completed every 700 ms during the measurements.

### 2.2. Odorants

The odorants included in the present study were sulfur compounds, carboxylic acids, trimethylamine, and 3-methylphenol. The compounds included in the present study are either considered to be some of the most significant odorants or are found in high concentrations in air from pig production [10,13,19–21]. 3-Methylphenol was used as a surrogate for 4-methylphenol which is normally found in the air from pig production facilities, however the chemical properties are the same and the results should be transferable. The reference gas, *n*-butanol, was also included in the study since this compound is used to select panelists for olfactometry. The odorants were introduced into the olfactometer as single compounds. Certified hydrogen sulfide, methanethiol, *n*-butanol (all from AGA, Copenhagen, Denmark), dimethyl sulfide and trimethylamine (all from Air Liquide, Horsens, Denmark) were introduced from pressurized gas cylinders. The gas cylinder containing *n*-butanol had an initial concentration at 68.2 ppm_v_ and was prediluted with atmospheric air purified by a Supelpure HC filter (Supelco, Bellefonte, PA, USA). Acetic acid, propanoic acid, butanoic acid, 3-methylphenol were generated from permeation tubes (VICI Metronics, Inc., Houston, TX, USA) using a permeation oven (Dynacalibrator model 150, VICI Metronics Inc.). The permeation tubes were calibrated gravimetrically prior to the measurements. The flow of the odorant mixtures and the predilution of *n*-butanol were controlled by mass flow controllers (Bronkhorst, Ruurlo, The Netherlands). The gas dilution system (reduction valve/permeation oven, mass flow controllers, and tubing) was allowed to equilibrate for at least one hour before the measurements were carried out.

### 2.3. Measurements

**Figure 1.**
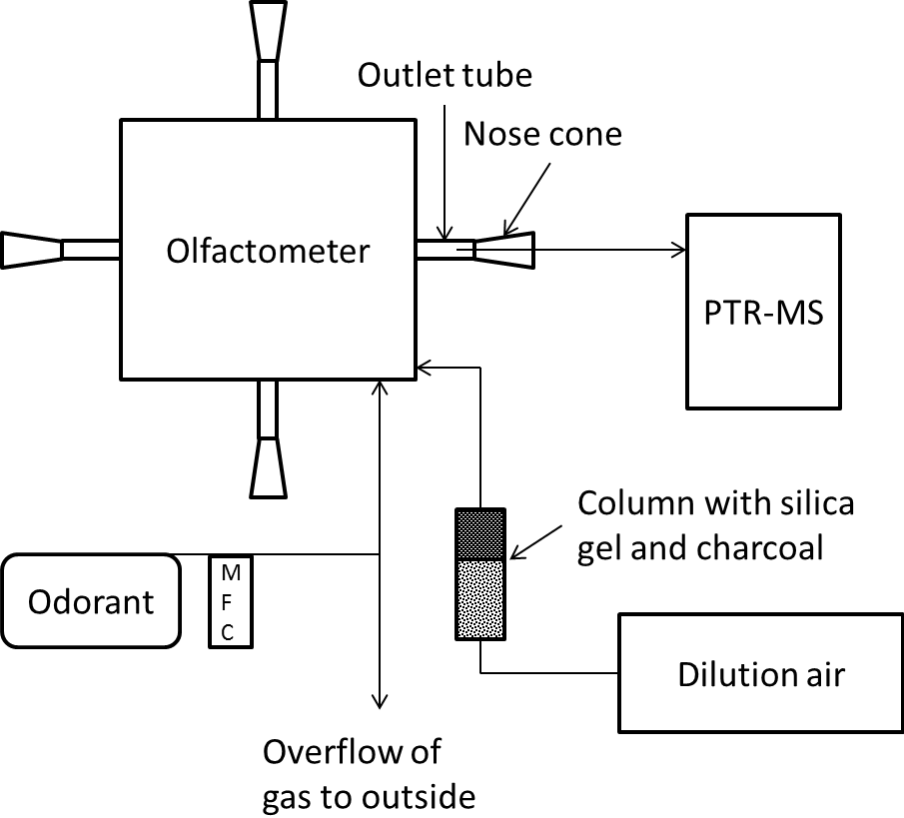
Schematic drawing of the experimental setup.

Gas samples with the single odorants were diluted in the olfactometer in two sessions where the concentration was: (i) measured in the outlet tubing from the olfactometer, or (ii) in the centrum of a nose cone where the human nose is normally placed, see Figure 1. The measurements were performed in one of the four sniffing ports, but the agreement between them was checked prior to the measurements. The odorant mixtures were diluted in decending order from 4,096 to 128 times dilution with a step factor of two. Between each dilution step there was a blank sample only with dilution air. The pulse duration was set at 60 s which is the maximum for this specific olfactometer. The recovery of odorants in the olfactometer at each dilution step was estimated based on the last ten measurement cycles of the pulse (∼6 s) relative to the expected concentration (defined as the undiluted sample concentration measured by PTR-MS divided by the dilution factor). The response time of the PTR-MS was estimated based on the PTR-MS sample line recovery when changing from a highly diluted sample (>30 times) to an undiluted sample after conditioning the system for one h. The PTR-MS response time was defined as the time to reach 90% of the final concentration.

## 3. Results and Discussion

In Table 1 the measured concentrations of the nine odorants in the undiluted samples are presented along with the detection limits for the PTR-MS and the measured concentration at each dilution step. The concentration levels for the undiluted samples were chosen to be in the ppm_v_-range in order to achieve concentrations above the detection limit for the PTR-MS after dilution in the olfactometer. It can be seen from Table 1 that except for trimethylamine at the highest dilution steps the measured concentrations were above the detection limit for the PTR-MS. The relative standard deviation was higher at the highest dilution levels (10–30%) compared to the lowest dilution levels (<10%) and this has to be taken into consideration when the results are evaluated.

### 3.1. Recovery of Odorants in the Olfactometer

**Figure 2.**
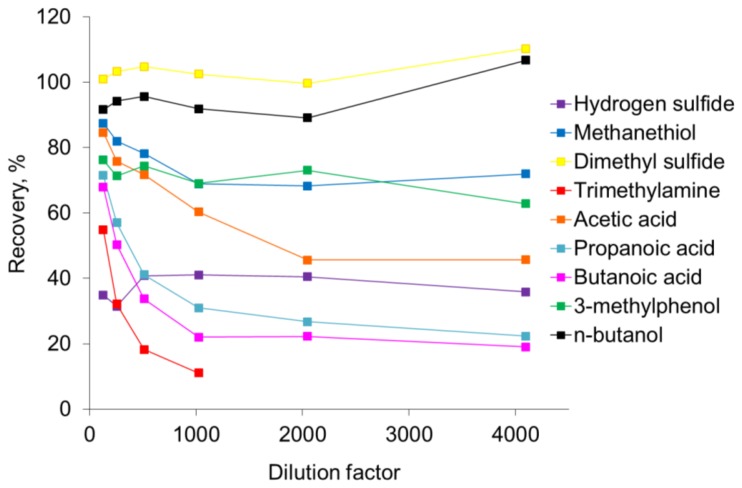
Recovery of odorants in the outlet tubing of an olfactometer as a function of the dilution factor.

The recovery of odorants in the olfactometer is presented in Figure 2. The recovery data in Figure 2 represents the average of the measurements in the outlet tubing from the olfactometer (last ten cycles ∼6 s out of 60 s) relative to the expected concentration (defined as the undiluted sample concentration measured by PTR-MS divided by the dilution factor). Dimethyl sulfide and *n*-butanol were only slightly affected by the dilution in the olfactometer. Dimethyl sulfide was almost completely recovered, whereas the recovery of *n*-butanol was 90–95%. The odorant *n*-butanol is used to select panelists for olfactometry [1], and the high recovery of this odorant demonstrates that the olfactometer only has a limited influence on the panel selection. The recovery of carboxylic acids (acetic acid, propanoic acid and butanoic acid) and methanethiol and trimethylamine were significantly affected by the dilution in the olfactometer. It seems that the recovery of these compounds depends on the dilution level where the recovery increased as the dilution level was lowered. For the carboxylic acids the recovery was 20–50% at the highest dilution level and 70–85% at the lowest dilution level. Trimethylamine was below the detection limit at the two highest dilution levels (4,096 and 2,048 times dilution) and the recovery could not be estimated. However, based on the recovery seen for trimethylamine at the other dilution levels the recovery may only be a few percent. Methanethiol was less affected by the dilution in the olfactometer compared to the carboxylic acids and trimethylamine with a recovery at *ca.* 70% at the highest dilution level and *ca.* 85% at the lowest dilution level. The recoveries of hydrogen sulfide (35–45%) and 3-methylphenol (65–75%) were also affected by the dilution in the olfactometer, but it seems that these odorants were less affected by the dilution level. However, it has to be stated that the concentration level used in the present study was in the ppm_v_-range and in air from pig production the concentration level will normally be in the ppb_v_-range. Consequently, the recovery of compounds in an air sample from pig production facilities is likely to be in the range of the highest dilution levels in the present study.

### 3.2. Development in the Recovery of Odorants during a 60-Second Pulse

**Figure 3.**
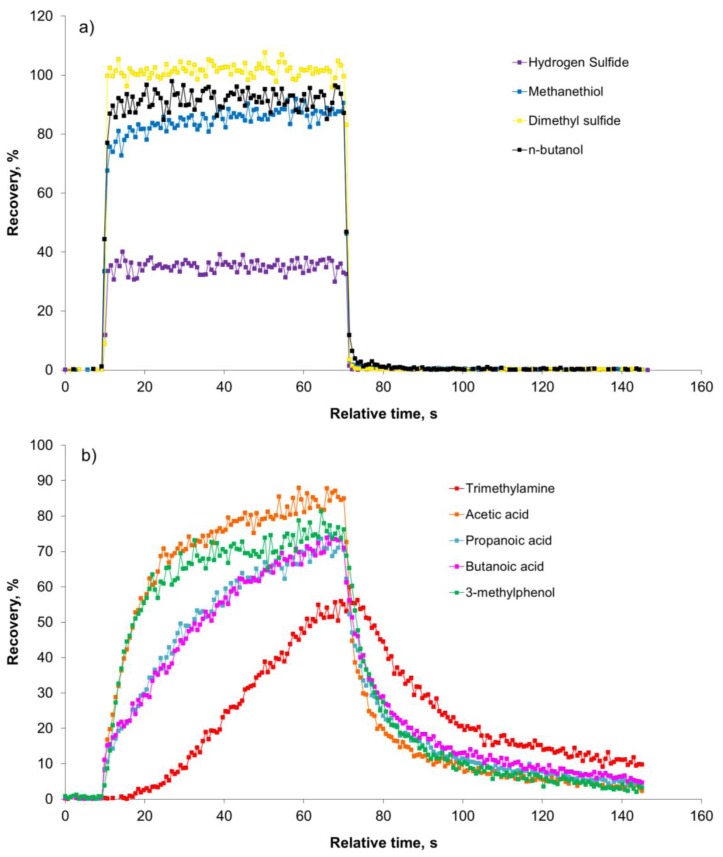
Development in the recovery during a 60-second pulse for selected odorants in an olfactometer. The odorants were introduced to the olfactometer after 10 s and the pulse stopped after 70 s.

In Figure 3 the development in the recovery of the nine odorants during a 60-second pulse is shown for the lowest dilution level (128 times dilution).

**Figure 4.**
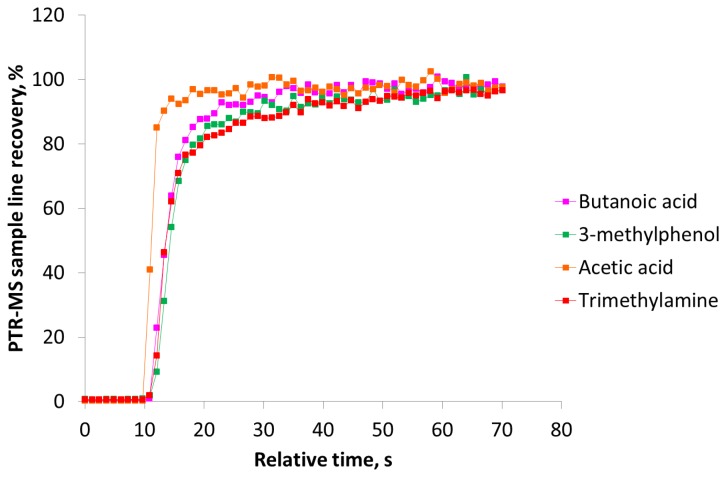
PTR-MS response time for individual odorants measured with a 1.2 m PEEK sampling line heated to 60 °C. The PTR-MS response time was defined as the time to reach 90% of the final concentration when changing from a highly diluted sample to an undiluted sample (relative time = 10 s).

Figure 3(a) clearly demonstrates that the sulfur compounds and *n*-butanol reach the maximum recovery within a few seconds and return to the background level immediately after the pulse. In a study with an olfactometer for medical purposes constructed of PTFE the maximum signal of hydrogen sulfide was achieved after 3.2 s [8]. In our study the maximum recovery of hydrogen sulfide was reached after *ca.* 2 s. According to Figure 3(b) the recovery of carboxylic acids, trimethylamine and 3-methylphenol is increasing during the entire pulse without reaching a stable level. A part of the delay could be due to delay in the sample line of the PTR-MS (1.2 m PEEK tubing, outer diameter 1.6 mm, inner diameter 0.064 mm, and heated to 60 °C) and this has to be taken into consideration, which was done in the next series of measurements. In Figure 4 the estimated PTR-MS response time is demonstrated for two of the carboxylic acids, trimethylamine and 3-methylphenol. The PTR-MS response time was defined as the time to reach 90% of the final concentration. The PTR-MS response time for acetic acid is below 5 s, for butanoic acid and 3-methylphenol it is between 10–15 s and for trimethylamine it is *ca.* 25 s. This means that except for trimethylamine the recovery after 15 s is mainly due to the effect of the olfactometer. According to the European standard for dynamic olfactometry [1] the pulse duration can be a maximum of 15 s and in practice it is often only a few seconds. Consequently, in practice the recovery of some odorants will be lower than the estimated recovery after 60 s (Figure 2). Depending on the chemical composition of the air matrix of interest, the pulse duration can in some cases have a large influence on the estimated odor concentration. It seems that the maximum pulse duration at 15 s is insufficient to achieve a high recovery of certain odorants when air from pig production is analyzed and either the pulse duration should be increased or the recovery of odorants in the dilution system should be increased.

The recovery of the compounds included is not a straightforward function of volatility. For example, no correlation is observed between the recovery at a dilution factor of 1024 and the logarithm of the compound vapor pressure at room temperature (data not shown; R^2^ = 0.003) even when excluding trimethylamine (data not shown; R^2^ = 0.03), which appears to be an extreme case. Thus, the poor recovery is most likely due to a combination of factors, including polarity, volatility, hydrogen donor abilities (OH groups, SH groups) and hydrogen acceptor abilities (nitrogen compounds). It is noteworthy that the best recovery is seen for dimethyl sulfide, which is a relatively non-polar compound, whereas the poorest recovery is seen for trimethylamine, which is a polar hydrogen acceptor with a higher vapor pressure than dimethyl sulfide [22]. From Figure 3(a) it seems clear that the reduced recovery of H_2_S, methanethiol and *n*-butanol is mainly due to a reactive removal process, since the mass missing in the outlet during loading is not recovered, when the pulse is stopped. For the carboxylic acids, trimethylamine and 3-methylphenol in Figure 3(b) the reduced recovery is most likely due to a combination of reactive removal processes and adsorption to surfaces in the dilution system since the compounds are recovered to some extent after the pulse is stopped. In the present study the parts of the olfactometer in contact with the sample air were made of Teflon (tubing) and stainless steel (orifices). In previous studies regarding sample introduction to sample bags it has been demonstrated that Teflon is a relatively inert material in relation to sulfur compounds [23,24], whereas stainless steel results in a loss [23]. The inertness of Teflon and stainless steel in relation to other odorants has not been investigated, but it is likely that an equilibration time is necessary and this may be critical when the pulse duration is short. The need for an equilibration time may also explain the improved recovery as the dilution level decreases.

### 3.3. Effect of the Nose Cone on the Measured Concentration

**Figure 5.**
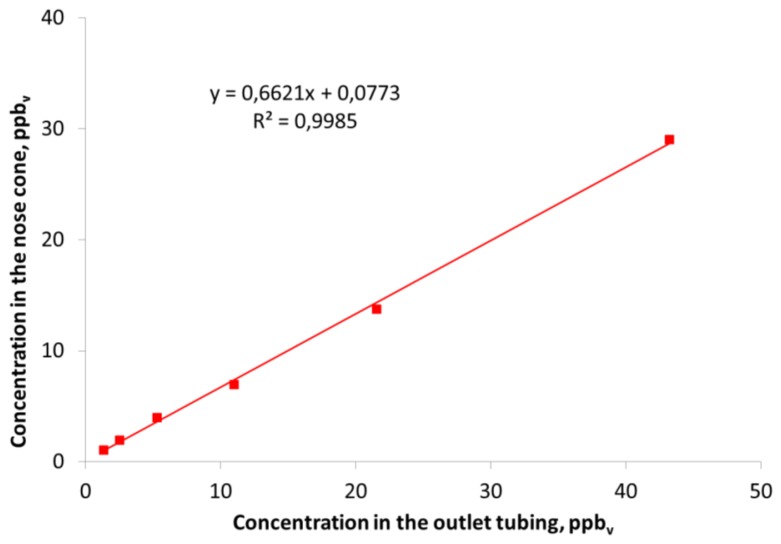
Correlation between the concentration of dimethyl sulfide measured in the outlet tubing from the olfactometer and in the nose cone where the human nose is placed.

All the previous results that have been presented are concerned with measurements in the outlet tubing from the olfactometer without considering any effect from the nose cone. In Figure 5 the correlation between the measured concentration in the outlet tubing of the olfactometer and in the center of the nose cone is shown for dimethyl sulfide.

Figure 5 clearly demonstrates that the concentration measured in the centrum of nose cone where the human nose is placed during measurements was lower compared to the concentration measured in the outlet tubing. The same trend was also seen for the other compounds that were included in the study (results not shown). Dimethyl sulfide was almost completely recovered from the outlet tubing of the olfactometer and the lower recovery (70–80%) in the nose cone can mainly be ascribed to the cone itself. In a previous study with the same type of olfactometer a recovery of dimethyl sulfide at *ca.* 80% was also measured in the nose cone [7]. Smoke from an air flow indicator tube was used to investigate the flow patterns around the nose cone when a sample was diluted in the olfactometer (results not shown). It was demonstrated that the air inside the nose cone was diluted with the surrounding air due to the shape of the nose cone. The conical shape of the nose cone creates turbulence inside the nose cone which causes an air flow into it. During real measurements the human nose will be placed inside the nose cone and this dilution may be reduced. However, it requires that all panelists use the nose cone in the same way otherwise this dilution with surrounding air may influence the results. An alternative could be to use a cylindrical nose cone instead of the conical nose cone which could diminish the dilution with surrounding air.

## 4. Conclusions

It can be concluded from the present study that an olfactometer designed according to the European standard for olfactometry can significantly influence the recovery of odorants such as sulfur compounds, carboxylic acids, trimethylamine and 3-methylphenol. The final recovery of carboxylic acids, trimethylamine and 3-methylphenol was highly dependent on the pulse duration and did not reach a stable level during a 60-second pulse. The sulfur compounds and *n*-butanol reached a stable signal within a few seconds, but only dimethyl sulfide reached the expected level (∼100% recovery). Thus, differential recovery of odorants can lead to a different compound composition compared to the sample that is introduced to the olfactometer. The results of the present study underlines that the design of the olfactometer (e.g., materials in contact with the sample and design of nose cones) and the operation management (e.g., pulse duration) are important parameters that has to be included in the future development of olfactometry.

## Figures and Tables

**Table 1.** Concentrations of odorants prior to dilution in the olfactometer and the concentrations measured at each dilution step by PTR-MS. The expected concentrations are shown in brackets.

**Compound**^1^	**H_2_S**	**MT**	**DMS**	**TMA**	**AA**	**PA**	**BA**	**3MP**	**NB**
*m*/*z*^2^	35	49	63	60	61	75	89	109	57
DL^3^, ppb_v_	0.2	0.02	0.1	0.2	0.1	0.04	0.1	0.2	0.2
Undiluted sample, ppm_v_	5.0	5.2	5.4	5.0	5.7	4.3	5.1	3.2	5.0

Dilution factor	ppb_v_ ± 1 SD								

4,096	0.5 ± 0.2 (1.2)	0.9 ± 0.1 (1.3)	1.5 ± 0.2 (1.3)	<0.2 (1.2)	0.6 ± 0.1 (1.4)	0.2 ± 0.1 (1.1)	0.2 ± 0.1 (1.3)	0.5 ± 0.1 (0.8)	1.3 ± 0.3 (1.2)
2,048	1.1 ± 0.2 (2.4)	1.7 ± 0.1 (2.5)	2.6 ± 0.2 (2.7)	<0.2 (2.5)	1.3 ± 0.2 (2.8)	0.6 ± 0.1 (2.1)	0.6 ± 0.1 (2.5)	1.2 ± 0.2 (1.6)	2.2 ± 0.3 (2.4)
1,024	2.1 ± 0.4 (4.9)	3.5 ± 0.3 (5.0)	5.4 ± 0.3 (5.3)	0.5 ± 0.2 (4.9)	3.3 ± 0.3 (5.5)	1.3 ± 0.1 (4.2)	1.1 ± 0.1 (5.0)	2.2 ± 0.2 (3.2)	4.5 ± 0.5 (4.9)
512	4.1 ± 0.6 (9.7)	7.9 ± 0.6 (10)	11 ± 0.4 (11)	1.8 ± 0.2 (9.9)	7.9 ± 0.4 (11)	3.5 ± 0.2 (8.5)	3.4 ± 0.2 (10)	4.7 ± 0.4 (6.3)	9.3 ± 0.4 (9.8)
256	6.2 ± 0.5 (19)	16 ± 0.7 (20)	22 ± 0.5 (21)	6.3 ± 0.4 (20)	17 ± 0.5 (22)	9.7 ± 0.3 (17)	10 ± 0.3 (20)	9.0 ± 0.4 (13)	18 ± 0.5 (20)
128	14 ± 1 (39)	35 ± 0.7 (40)	43 ± 1 (42)	22 ± 1 (39)	37 ± 0.9 (44)	24 ± 0.7 (34)	27 ± 0.6 (40)	19 ± 0.6 (25)	36 ± 1 (39)

1 H_2_S: hydrogen sulfide; MT: methanethiol; DMS: dimethyl sulfide; TMA: trimethylamine; AA: acetic acid; PA: propanoic acid; BA: butanoic acid; 3MP: 3-methylphenol; NB: *n*-butanol;

2 *m*/*z*: mass-to-charge ratio;

3 DL: Detection limit estimated as three times the standard deviation on a blank sample (dry air).
